# The developmental origins of heterodonty and acrodonty as revealed by reptile dentitions

**DOI:** 10.1126/sciadv.abj7912

**Published:** 2021-12-17

**Authors:** Lotta Salomies, Julia Eymann, Joni Ollonen, Imran Khan, Nicolas Di-Poï

**Affiliations:** Institute of Biotechnology, Helsinki Institute of Life Science, University of Helsinki, 00014 Helsinki, Finland.

## Abstract

Despite the exceptional diversity and central role of dentitions in vertebrate evolution, many aspects of tooth characters remain unknown. Here, we exploit the large array of dental phenotypes in acrodontan lizards, including *EDA* mutants showing the first vertebrate example of positional transformation in tooth identity, to assess the developmental origins and evolutionary patterning of tooth types and heterodonty. We reveal that pleurodont versus acrodont dentition can be determined by a simple mechanism, where modulation of tooth size through EDA signaling has major consequences on dental formula, thereby providing a new flexible tooth patterning model. Furthermore, such implication of morphoregulation in tooth evolution allows predicting the dental patterns characterizing extant and fossil lepidosaurian taxa at large scale. Together, the origins and diversification of tooth types, long a focus of multiple research fields, can now be approached through evo-devo approaches, highlighting the importance of underexplored dental features for illuminating major evolutionary patterns.

## Teaser

Analysis of reptile models reveals the developmental origins and diversification of tooth identity in extant and fossil taxa.

## INTRODUCTION

Teeth are among the most distinctive features of vertebrates, thanks to their exceptional diversity. Tooth complexity is generally strongly correlated to dietary habits, life history, or ecological adaptations across species, justifying why dental morphological characteristics such as shape and size are commonly used to identify patterns of vertebrate evolution. Similarly, given the advantages of teeth as developmental models of organogenesis, the signaling pathways and morphogenetic processes explaining variation in dental morphology have been the focus of intense investigation over the past few decades (*1*). This allowed the building of models for predicting evolutionary patterns of teeth at least within mammals (*2*). Tooth development is a highly conserved process, proceeding through similar stages and using the same set of genetic factors in all examined species (*1*). One of the most closely studied signaling pathways associated with changes in dental morphology is the ectodysplasin (EDA) pathway, which regulates the development of various ectodermal organs, including teeth, across vertebrates (*3*). In mammals, EDA signaling influences tooth shape and positively regulates tooth size and number. Furthermore, changes in this pathway have been implicated in driving evolutionary change in murine teeth (*4*), suggesting a key role in shaping vertebrate dentitions.

**Fig. 1.**
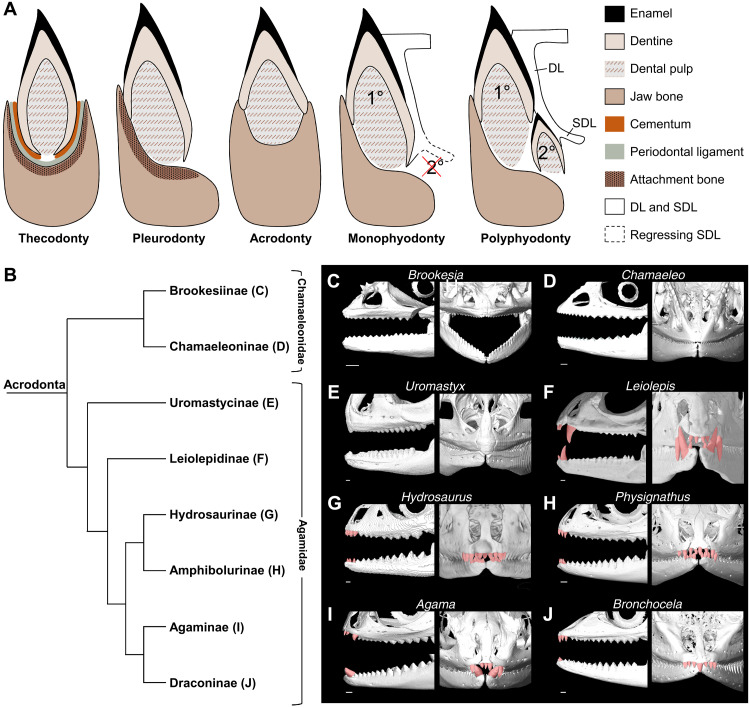
Phylogeny and tooth diversity of Acrodonta. (**A**) Schematic images describing some of the different tooth attachment types and tooth replacement patterns present in vertebrates. Thecodont teeth lie in sockets and are attached via a periodontal ligament, while pleurodont and acrodont teeth are directly fused to the jaw bone via ankylosis. Monophyodont teeth are not replaced, and the SDL is usually not maintained. Polyphyodont teeth maintain a SDL and are continuously replaced. DL, dental lamina; SDL, successional DL; 1°, first-generation tooth; 2°, second-generation (replacement) tooth. (**B**) Phylogenetic tree showing main families and subfamilies of Acrodonta, adapted from the most inclusive phylogenetic study available for extant squamates (*55*). (**C** to **J**) 3D-rendered adult skulls of selected lizards representative of all main Acrodonta subfamilies, highlighting the various patterns of jaw teeth in lateral (left panels) or frontal (right panels) views: *Brookesia brygooi* (C), *Chamaeleo calyptratus* (D), *Uromastyx hardwickii* (E), *Leiolepis belliana* (F), *Hydrosaurus amboinensis* (G), *Physignathus cocincinus* (H), *Agama hispida* (I), and *Bronchocela jubata* (J). The frontal view in (G) is a volume rendering for optimal image quality, whereas all other images are skull or jaw segmentations. Anterior pleurodont teeth are highlighted in red when present (F to J), and acrodont teeth are noncolored. Scale bars, 1 cm.

The laboratory mouse is a classic mammalian system in which many key mechanisms and molecular regulation of odontogenesis have been identified. However, the mouse model has a highly derived and reduced dentition, even compared with the situation in other mammals, and has some limitations. First, the dentition only comprises anterior incisors and posterior molars separated by a toothless region. Second, the dentition only has one set of teeth, none of which are replaced during the animal’s lifetime (monophyodonty; Fig. 1A). Third, all teeth show a single type of major dental implantation mode where the tooth sits within a socket in the jaw bone (thecodonty; Fig. 1A) (*5*). As a consequence, in particular given the lack of canines and premolars and, subsequently, tooth replacement, the mouse is not the best model to understand tooth evolution and development in mammals. Furthermore, most nonmammalian taxa display dental features contrasting from the mouse, except for thecodonty, which has been identified in crocodilians and many extinct archosaurs (*6*, *7*). Notably, continuously replaced teeth (polyphyodonty) having no roots and being attached to the lateral side of the jaw bone (pleurodonty) are relatively common in vertebrates (Fig. 1A) and considered the primitive condition in diverse amniote and anamniote lineages, both extinct and extant (*8*–*10*). Furthermore, another major type of dental implantation, where teeth are fused to the apex of the jaw without sockets (acrodonty; Fig. 1A), is exclusively represented in some extinct and extant reptiles (*10*–*17*) as well as in anamniotes such as bony fish and lissamphibians (*8*, *18*). As a result, despite the long-standing focus on the characterization of morphological diversity and embryonic patterning in mammalian teeth, the molecular basis of developmental mechanisms underlying evolutionary patterning and variation in key dental features such as tooth implantation and regeneration capacity remains relatively understudied in vertebrates. More notably, the developmental processes at the origin of tooth identity and variation in tooth classes are still largely unknown.

The confirmed value of dental formula in multiple evolutionary developmental biology (evo-devo) research aspects, ranging from phylogenetic, taxonomic, and ecological identification of extant and extinct species to elucidation of fundamental principles of organogenesis and phenotypic variation and regenerative medicine, highlights the potential of increased knowledge on underexplored dental features. Here, we assess the developmental mechanisms underlying the formation and evolutionary changes in tooth identity and dental formula by exploiting the extraordinary phenotypic diversity of reptiles including a unique mutant lizard model showing the first vertebrate example of positional change in tooth identity. We report that the dentition patterns can be determined by a simple and flexible morphoregulatory process involving EDA signaling, a well-conserved pathway involved in the development of vertebrate skin appendages such as teeth (*3*, *4*), thus providing new perspectives on vertebrate dental patterning. Furthermore, we show that experimental data on nonmammalian dentitions can be extrapolated to fossil and extant taxa, particularly in some key vertebrate groups with a long evolutionary history and/or poor fossil record, thus allowing the prediction of the developmental origins, patterns, and evolutionary transitions in vertebrate dentitions beyond mammals.

## RESULTS

### Phenotypic diversity and implantation mode in lepidosaurian dentitions

Lepidosaurian reptiles, including Squamata (lizard and snakes) and Rhynchocephalia (tuatara and extinct relatives), are extremely diverse and represent ideal model systems to assess major aspects of tooth diversity found in nonmammalian lineages. In this group, species represent a multitude of ecologies and show a large array of dental phenotypes, ranging from simple conical to complex multicuspid teeth, directly reflecting dietary specialization (*19*). Some lepidosaurs also bear atypical heterodont dentitions, signifying that they bear teeth with different shapes or implantation modes in different parts of the skull and/or jaw, while others are homodont, having only one tooth type (*15*, *18*, *20*, *21*). Notably, while pleurodonty is highly common in snakes and lizards and accepted as the ancestral state for Squamata and Lepidosauria as a whole (*10*, *22*, *23*), teeth of certain taxa such as acrodontan iguanians (Agamidae and Chamaeleonidae), amphisbaenians (Trogoniphidae), and the tuatara display an understudied acrodont implantation or a mix of pleurodont and acrodont attachment (Fig. 1A) (*11*, *13*, *15*, *24*). A reduced or lack of tooth replacement is typically associated with acrodonty in living lepidosaurs (*11*), and the fossil record indicates that this condition is a highly derived feature of this group (*10*, *14*). As a result, in addition to variation in tooth shape and location, dental implantation and regeneration potential are much more diversified in Lepidosauria than in other amniote groups such as Crocodilia or Mammalia.

Our three-dimensional (3D) computed tomography (CT) analysis of dentitions in representative species of all extant families and subfamilies of Acrodonta lizards reflects particularly well this rich tooth diversity (Fig. 1B and table S1). Members of the Chameleonidae family are fully homodont, having only acrodont and monophyodont teeth symmetrically ankylosed to the mandibular and maxillary bones (Fig. 1, C and D). In contrast, Agamidae show a wide range of heterodont phenotypes, including variation in the size, shape, and number of acrodont and pleurodont teeth at posterior and anterior positions, respectively (Fig. 1, E to J). As previously described in osteological studies, the number of pleurodont teeth in agamids usually varies from 9 in *Agama* to more than 15 in *Hydrosaurus* (*11*–*13*), although all teeth are not necessarily visible simultaneously because of their polyphyodont phenotype (Fig. 1, G and I). A notable exception is *Uromastyx*, which apparently lacks polyphyodont teeth and shows extreme tooth wear at anterior positions, a phenotype largely associated with particular ecological life habits (Fig. 1E) (*25*). Agamids also show high diversity in pleurodont tooth size, ranging from the relatively small teeth in *Bronchocela* (Fig. 1J) to the very large, canine-like pleurodont teeth in *Leiolepis* (Fig. 1F). Last, some variation exists in acrodont tooth shape, with some species like *Physignathus* and *Bronchocela* having a tricuspid pattern with lateral cusps (Fig. 1, H and J), while others like *Agama* showing simple triangular, unicuspid teeth (Fig. 1I).

### New insights into dental patterning provided by EDA signaling in lizards

To elucidate the developmental mechanisms of evolutionary change in lepidosaurian dentition, we took advantage not only of the extraordinary phenotypic diversity of Acrodonta but also of the existence of established wild-type and spontaneous mutant research models in this group (*26*–*28*). More specifically, we characterized the yet unexplored function in dental tissues of EDA, a well-conserved signaling pathway crucial for the developmental patterning and evolutionary origin of ectodermal appendages across vertebrates (*3*, *25*, *29*–*35*). We initially focused on the agamid bearded dragon (*Pogona vitticeps*), which shows an intermediate heterodont phenotype (11 pleurodont teeth) within Acrodonta and represents an emerging model for various aspects of vertebrate dentitions (*13*, *36*).

**Fig. 2.**
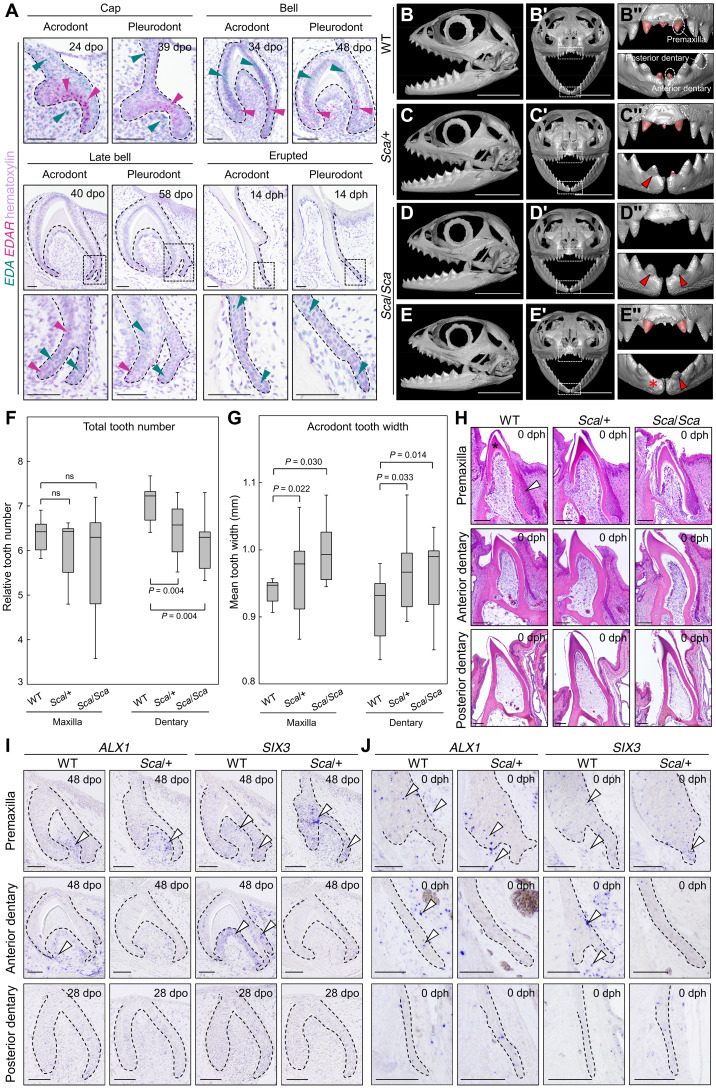
Characterization of mutant scaleless *P. vitticeps* dentition. (**A**) RNAscope in situ hybridization (ISH) of *EDA* (green) and *EDAR* (magenta) expression during odontogenesis in acrodont and pleurodont dentitions of wild-type (WT) *P. vitticeps*. Tissues are counterstained with hematoxylin (purple). Developmental stages are indicated as embryonic days postoviposition (dpo) or days posthatchling (dph). High magnifications of the SDL are shown for late stages (bottom panels, dashed boxes). Green and magenta arrowheads indicate regions of *EDA* and *EDAR* expression, respectively. (**B** to **E**″) 3D-rendered skulls of littermate WT, heterozygous scaleless (*Sca*/+), and homozygous scaleless (*Sca*/*Sca*) lizards at 14 dph in lateral (B to E) and frontal (B′ to E″) views. (B″) to (E″) show close-ups of dashed boxes in (B′) to (E′). The main WT tooth types based on their location are indicated in (B″). Morphologically normal pleurodont teeth are highlighted in red, whereas large, acrodont-like mutant teeth at the anteriormost dentary position are indicated by red arrowheads. The red asterisk indicates an empty tooth position. (**F** and **G**) Box plots depicting the relative tooth number (F) or acrodont tooth width (G) in maxillary and dentary bones of WT, *Sca*/+, and *Sca*/*Sca* at early postnatal stage. Significant (*P* < 0.05) and nonsignificant (ns) values are indicated. (**H**) Hematoxylin and eosin–stained sections of hatchling WT, *Sca*/+, and *Sca*/*Sca* teeth from indicated positions, as visualized in (B″). The locations of the DL (arrowhead) and dentin (asterisk) are indicated in the top left panel. (**I** and **J**) ISH of *ALX1* and *SIX3* at bell stage (I) and in the SDL of erupted hatchling teeth (J) in WT and *Sca*/+ at indicated dental positions. The epithelium-mesenchyme junction is indicated by black dashed lines. White arrowheads indicate detection of gene expression in all premaxillary teeth and WT anterior dentary teeth only. Scale bars, 50 μm (A and I to J), 5 mm (B to E″), and 100 μm (H).

As an important step to investigate the role of EDA, we first characterized the expression pattern of *EDA* and its receptor *EDAR* during *P. vitticeps* odontogenesis by double in situ hybridization (ISH) labeling with RNAscope assay (Fig. 2A). Despite the developmental delay of pleurodont versus acrodont dentition (*36*), a robust expression of *EDA* is observed in the outer enamel epithelium (OEE) and dental lamina (DL) in both types of teeth at “cap” and “bell” developmental stages, similarly to the pattern observed in mouse molars of equivalent stage (*37*). In contrast, *EDAR* localizes to the inner enamel epithelium (IEE), although its distribution is more widespread toward the cervical loops than in mouse. Since EDA is a key regulator of tooth shape evolution across vertebrates (*3*, *4*), this differential expression pattern could potentially contribute to some differences between mouse and lizard dental morphology (*19*, *38*). Additional *EDA* expression sites not previously described include the mesenchymal condensation and IEE at cap and bell stages, respectively. Starting from “late bell” stage, *EDA* and *EDAR* remain expressed at lower levels in OEE/IEE and cervical loop compartments, respectively, and both genes are weakly detected in the forming successional DL (SDL). Only *EDA* remains expressed in the SDL of both tooth types at later developmental stages. Of note, although no major differences in the expression levels and dynamics were noticed between acrodont and pleurodont teeth, a weak expression of *EDA* is detectable in the mesenchyme surrounding the SDL of pleurodont teeth only (Fig. 2A, insets), coherent with the differential patterning of the developing SDL structure in both dentitions (*36*).

On the basis of this expression pattern of key components of the EDA pathway, we next assessed the function of EDA signaling by exploring the yet uncharacterized dental phenotype of unique scaleless mutant *P. vitticeps* bearing a codominant mutation in *EDA* (*26*). To avoid any interindividual phenotypic variation associated with indeterminate growth, extensive acrodont tooth wear, and pleurodont tooth replacement in adult *P. vitticeps*, we primarily compared wild-type and mutant teeth with 3D CT (Fig. 2, B to E, and fig. S1A). At hatchling and early postnatal stages, the dentition in wild-type lizards consists of eight acrodont teeth and one relatively small, conical pleurodont tooth per jaw quadrant. At this early stage, anterior pleurodont teeth only locate to the dentary and premaxillary bones, and the central egg tooth on the premaxilla is shed and replaced by a new pleurodont tooth soon after hatchling (Fig. 2, B to B″). In contrast, both heterozygous (*Sca/+*) and homozygous (*Sca/Sca*) scaleless animals share an abnormal tooth formula partially associated with the frequent absence of premaxillary pleurodont teeth, resulting in about half of juvenile mutants with a mostly tooth-free premaxilla (Fig. 2, C to E″, and fig. S1, A to C). Furthermore, our quantification of the relative number of acrodont teeth on the different jaw quadrants indicates a significantly decreased tooth number in mutant animals, when compared with wild-type counterparts, a phenotype particularly pronounced on the dentary bones in *Sca/Sca* animals (Fig. 2, D to F). As expected from visual inspection of lateral skull views, this acrodont phenotype is not associated with differences in skull dimensions (fig. S1B). Instead, it appears directly linked to the increased width of mutant acrodont teeth relative to skull length, an effect more pronounced in homozygous mutants further indicating a dose-dependent effect (Fig. 2G).

Visual inspection of CT scans revealed another notable phenotype of the scaleless mutation. Both *Sca*/+ and *Sca*/*Sca* lizards show a relatively large size of the anteriormost tooth on one or two dentaries (Fig. 2, C to E″, and fig. S1A, red arrowheads). This contrasts with the small pleurodont teeth at corresponding positions in wild-type counterparts (Fig. 2B″ and fig. S1A). This size phenotype is highly penetrant in hatchling mutants (*n* = 18), affecting all examined *Sca*/*Sca* and about 66% of *Sca*/+ in at least one dentary, and its frequency is well correlated with the number of *Sca* alleles (fig. S1C). Indeed, whereas the majority of examined *Sca*/*Sca* and *Sca*/+ mutants have a symmetric phenotype, with one relatively large tooth on the anteriormost part of each dentary, a few specimens exhibit asymmetry with increased size or total absence of tooth only on one side (Fig. 2, C″ and E″, and fig. S1C). Of note, no asymmetry was detected on the hatchling premaxilla, with both pleurodont teeth being always present or absent simultaneously (fig. S1C). Together, we show that the loss of *EDA* results in several major dental phenotypes in *P. vitticeps*, including increased tooth size and/or reduced tooth count at both acrodont and pleurodont positions. These data contrast with *EDA*-deficient mouse models, which not only exhibit the opposite effect on incisor and/or molar size (*39*–*41*) but also show severe defects in tooth complexity (*42*–*44*) not observed at least based on CT inspection in our mutant lizards. Although the expression pattern of components of the EDA pathway are relatively well conserved in *P. vitticeps*, differences in *EDA* function and/or developmental mechanisms regulating tooth complexity, including the enigmatic reptilian enamel knot structure (*45*), might explain interspecies phenotypic variations. Furthermore, shape-related effects may simply not be the same in lizards and mammals because of the fundamental differences in dental structure (*38*).

### Evidence of anterior tooth transformation in *EDA*-deficient *P. vitticeps*

One particularly intriguing phenotype of scaleless *P. vitticeps* is the apparent homodont-like dentition related to the presence of relatively large, nonconical teeth along the whole dentary. In these mutants, even the anteriormost dentary tooth resembles the triangular form of more posterior acrodont teeth, suggesting a potential replacement or transformation of anterior tooth identity from pleurodont to acrodont (Fig. 2, D to D″, and fig. S1A, red arrowheads). To our knowledge, besides the extremely rare cases of molarization and premolarization of anterior teeth observed in humans (*46*), the incisor-molar transformation induced in mouse explants by inhibition of bone morphogenetic protein (BMP) signaling provides the only reported example of tooth identity changes in vertebrates (*47*). Furthermore, the latter mouse phenotype was more recently attributed to a result of the splitting of the incisor placode rather than a true transformation of tooth type (*48*).

To better define the tooth identity of mutant lizard dentition, we next compared different tooth types based on their location rather than implantation mode: “premaxillary” and “anterior dentary” teeth (corresponding to pleurodont positions in wild type) and “posterior dentary” teeth (acrodont position in wild type) (Fig. 2B″). We particularly focused on recently identified developmental characteristics that distinguish embryonic and postnatal acrodont versus pleurodont dentition in *P. vitticeps*, including tooth morphology, histology, and molecular patterning (*36*). The true nature of acrodont teeth such as symmetric ankylosis of fusion to the jaw bone involves late remodeling events at adult stage (*10*, *17*). Similarly, tooth replacement is relatively slow in *P. vitticeps* and not yet visible in early dentition (*36*), preventing its practical use as an identifier of tooth type. On the basis of histological stainings of tooth sections from the premaxillary region, although some developmental delay is noticeable in *Sca*/+ and *Sca*/*Sca* mutants, both wild-type and mutant teeth, when present, are relatively small and show asymmetrical growth of labial and lingual parts typical of pleurodont-like dentition (Fig. 2H) (*17*, *49*). Similarly, for all genotypes, posterior dentary teeth have a regular triangular, acrodont-like morphology, with relatively tall tooth crowns and a long, thin DL. In contrast, *Sca*/+ and *Sca*/*Sca* anterior dentary teeth are much taller and show distinct morphology and increased dentin formation, compared with wild-type. Furthermore, the mutant DL at this anterior position appears relatively long and thin, similar to the situation in more posterior dentary teeth, suggesting an acrodont-like phenotype (Fig. 2H).

To confirm the identity of mutant teeth at the molecular level, we took advantage of markers previously identified as differentially expressed between wild-type pleurodont and acrodont teeth, including *ALX1* and *SIX3* (*36*). Because of the difficulty of obtaining homozygous mutants from *P. vitticeps* breeding, we proceeded with mainly *Sca*/+ mutants, which show a high penetrance of the phenotype of interest (fig. S1C). Comparative gene expression analysis by ISH between different tooth types at mid-developmental stage (Fig. 2I) and in the SDL of erupted teeth (Fig. 2J) indicates similar high levels of *ALX1* and *SIX3* expression in wild-type premaxillary and anterior dentary teeth, when compared with posterior dentary dentition, thus confirming the pleurodont tooth phenotype at these positions (*36*). In contrast, while the expression of *ALX1* and *SIX3* in mutant premaxillary teeth is relatively similar to their wild-type counterparts, both genes are down-regulated in anterior dentary teeth from *Sca*/+ mutants, resembling the pattern from posterior dentary teeth (Fig. 2, I and J). Together, both morphological and molecular data indicate that *EDA*-deficient anteriormost dentary teeth exhibit transformed, acrodont-like features, a phenotype already apparent from mid-odontogenesis.

### Developmental origins of tooth identity in *P. vitticeps*

We next took advantage of wild-type heterodonty and the marked change of tooth identity in mutants to identify the developmental origins of acrodont and pleurodont tooth types in *P. vitticeps*. To determine the stage where the developmental shift in dental identity could occur, we first examined a minority of *Sca/Sca* animals showing a missing tooth at the anteriormost position (Fig. 2, E to E″, and fig. S2A). Using histological staining of dental tissues, these mutants only show an early DL structure at empty dental spaces, similar to the situation in interdental regions, strongly indicating a complete arrest of tooth development at DL stage around or prior tooth initiation. Of note, the alternative hypothesis of resorption of truncated tooth germs was excluded because of the relatively intact DL structure (fig. S2A). Furthermore, analysis by ISH of *EDA* and *EDAR* gene expression pattern at the DL/initiation stage confirms the expression of both genes in all tooth types (fig. S2, B and D), supporting the potential importance of EDA signaling starting from early tooth developmental stages. Even more interesting is the expression profile of *NF*Κ*BIA*, a direct indicator of EDA/nuclear factor κB (NF-κB) pathway activity (*50*), which shows relatively high expression, particularly in anterior dentary and premaxillary teeth, and asymmetric pattern toward the growing DL tip (fig. S2, B and D).

**Fig. 3.**
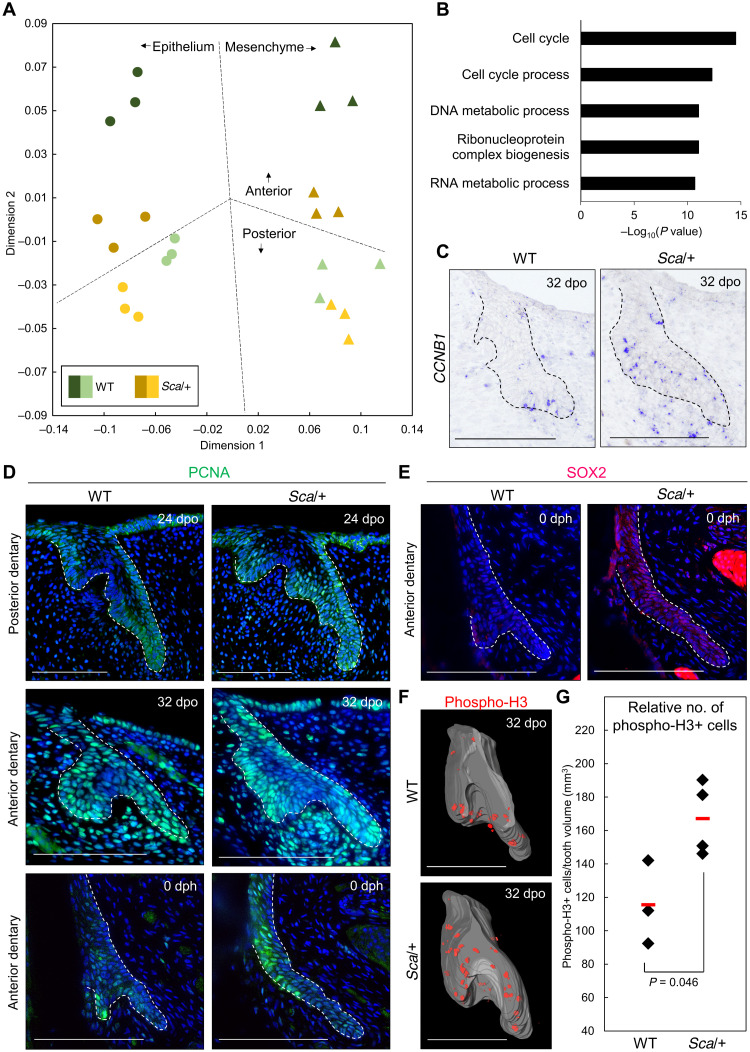
Characterization of mutant scaleless *P. vitticeps* dentition. (**A**) MDS analysis of transcriptomes from laser microdissected epithelial and surrounding mesenchymal tissues from WT and *Sca*/+ anterior and posterior teeth at DL/initiation stage. As shown in the plot, the colored circles and triangles represent epithelial and mesenchymal samples, respectively. WT samples are in green shades, and *Sca*/+ samples are in yellow shades; darker shades represent anterior samples, and lighter shades are posterior samples. (**B**) GO analysis of most significantly differentially regulated genes [−log_10_(*P* value) > 10] obtained from principal components (PC) analysis along the PC2 axis. (**C**) *CCNB1* expression on sections (ISH) from WT and *Sca*/+ anterior dentary tooth germs at the DL/initiation stage. The epithelium component is indicated by black dashed lines. (**D**) PCNA immunohistochemistry (IHC) in WT and *Sca*/+ posterior and anterior dentary teeth at the DL/initiation stage (top and middle) and in the SDL of erupted anterior dentary teeth (bottom). (**E**) SOX2 IHC in WT and *Sca*/+ SDL of erupted anterior dentary teeth. The sections are adjacent to the ones shown in the bottom of (D). Cell nuclei are counterstained with DAPI (blue staining), and the epithelium-mesenchyme junction is indicated by white dashed lines (D to E). (**F** and **G**) Reconstructed 3D models of phospho-H3 IHC (F) on WT and *Sca*/+ anterior dentary tooth germs at the DL/initiation stage and quantification of the data (G). The epithelium component in (F) is indicated by gray segmentation. In (G), the mean of the replicates (red bars) and *P* value are indicated in the panel. Scale bars, 100 μm (C to F).

On the basis of the phenotype of mutant lizards with aborted tooth formation, we further investigated the corresponding DL/initiation stage in wild-type and mutant teeth using a high-throughput, transcriptomic-based gene expression profiling approach. For this purpose, we performed staining-guided laser capture microdissection of DL epithelium and surrounding mesenchyme corresponding to wild-type and *Sca*/+ teeth at the anterior and posterior jaw positions, which we refer to as anterior and posterior tissues, respectively (Fig. 3A). The level of similarity of our overall transcriptome data was first assessed and visualized using multidimensional scaling (MDS) analysis. As expected, the transcriptomes of epithelial and mesenchymal tissues separate from each other along dimension 1, also confirming the accuracy of our laser microdissection protocol. Dimension 2 shows a clear segregation in both epithelial and mesenchymal compartments based on tooth location, particularly wild-type anterior (pleurodont) versus wild-type posterior (acrodont) tissues. Notably, however, such separation is not evident in *Sca*/+ mutants, where anterior tissues are positioned much closer to both *Sca*+ and wild-type posterior tissues corresponding to acrodont dentition. This transcriptome pattern further demonstrates the acrodont-like developmental program of mutant anterior tissues. To identify gene transcripts driving specific transcriptome segregation, we next performed a principal components (PC) analysis of the same original dataset (fig. S2C and table S2). We particularly focused on anterior- versus posterior-specific transcriptomes, which separate in wild-type tissues, but not in mutants, similarly to the MDS pattern. Gene ontology (GO) analysis of differentially expressed genes along PC2 shows significant enrichment of several GO terms related to cell cycle (Fig. 3B and table S2), indicating the importance of cell proliferation in the patterning of lizard dentition types. Of note, we found a relatively low number of classical tooth developmental genes significantly dysregulated between tissues and genotypes, in accordance with the importance of yet uncharacterized pathways in reptile dental tissues (*36*). In particular, with the exception of *SHH* up-regulation in epithelial *Sca*/+ tissues, no conserved EDA signaling target genes such as *FGF20* or Wnt/BMP inhibitors (*3*) show significant differential expression between mutant and wild-type tissues (table S2). Similarly, no significant differences were detected in EDA pathway genes (including *EDAR* and *EDARADD*) between genotypes, thus corroborating our ISH experiments (fig. S2D). These molecular data are coherent with the observed phenotypic differences between EDA-deficient mouse and lizard teeth. However, given its positive role in regulating tooth growth (*51*), the potential role of *SHH* in lizard dentition would require further investigation in the future, as this conserved gene could act downstream of EDA for regulating tooth size.

We next validated our transcriptomic approach by performing various analyses of cell proliferation markers during odontogenesis, with a particular focus on the different tooth identity phenotype observed at anteriormost dentary position in wild-type and *Sca*/+ teeth. We first assessed the expression pattern of conserved cell cycle–related genes such as cyclin B1 (*CCNB1*), one of the genes identified in our transcriptomic approach (table S2). As anticipated, CCNB1 is up-regulated in both epithelial and mesenchymal compartments of *Sca*/+ anterior dentary teeth at the DL/initiation stage, when compared with equivalent wild-type tissues (Fig. 3C). In accord with this, assessment by immunohistochemistry (IHC) of the expression pattern of proliferation marker proliferating cell nuclear antigen (PCNA) shows the expected rise of proliferating cells in *Sca*/+ sections of anterior dentary tooth germs (Fig. 3D). Such increase in cell proliferation is apparently not limited to mutant anterior teeth at the DL/initiation stage but also occurs in developing posterior dentary dentition. Furthermore, the increased proliferation pattern in mutant versus wild-type tissues persists and becomes even more pronounced at late developmental stages, including in the developing SDL of erupted teeth (Fig. 3D). Variations in DL and SDL structures were already noticed with histological stainings (see Fig. 2H), so we further validated molecular differences in SDL development by assessing the expression of the well-established dental stem cell marker SOX2 (*52*). As expected, an expanded SOX2 expression pattern is observed in *Sca*/+ SDL (Fig. 3E), indicating that increased cell proliferation is inherent to *EDA*-deficient tissues and not just a compensatory tooth scaling effect in response to reduced tooth number along the mutant jaw.

**Fig. 4.**
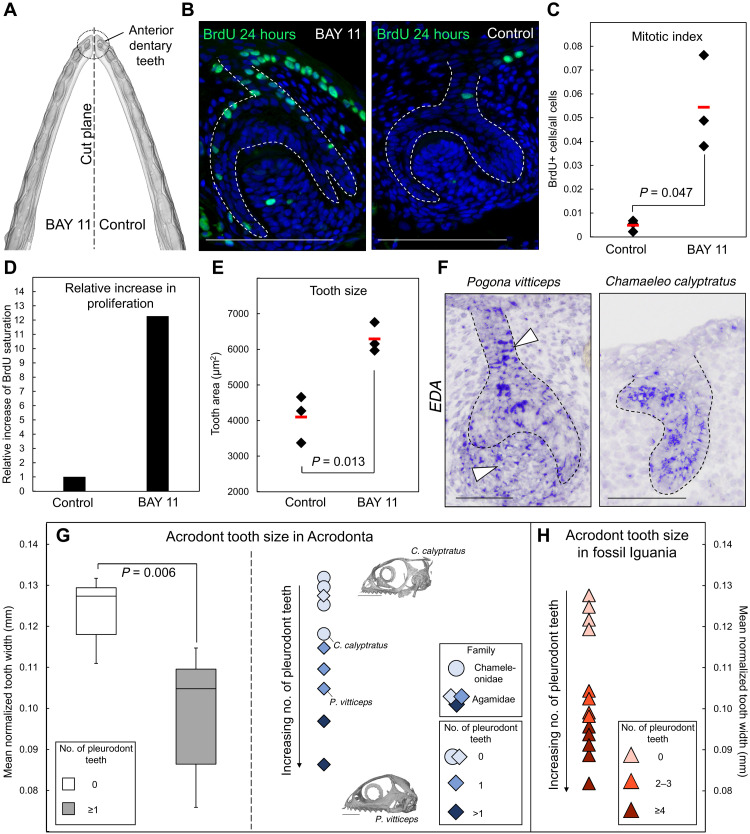
Proliferation pattern and size of lizard dentitions. (**A**) Schematic showing the ex vivo experimental setting used in (B) to (E). Left and right dentaries were separated and cultured for 7 days with either 20 μM EDA/NF-κB inhibitor (BAY 11) or ethanol (control), respectively. (**B**) BrdU IHC in BAY 11 or control tissues collected 24 hours after BrdU administration. The epithelium-mesenchyme junction is indicated by white dashed lines. (**C** to **E**) Plots depicting the number of proliferating cells divided by the total cell number (C), the relative increase in proliferation (D), and the tooth area (E) in three independent BAY 11 or control explants. The replicate mean (red bars) and *P* values are indicated in (C) and (E). (**F**) *EDA* ISH in acrodont dentary teeth at cap stage from *P. vitticeps* and *C. calyptratus* embryos. White arrowheads indicate increased *EDA* expression in *P. vitticeps* DL and dental papilla. (**G**) Combined plots depicting the normalized acrodont tooth size of selected extant Acrodonta at hatchling stage. The box plot (left) depicts tooth width in samples without (0) or with (greater than or equal to 1) pleurodont teeth in each jaw quadrant. The scatter plot (right) depicts tooth width in Chameleonidae (circles) or Agamidae (diamonds) species with different number of pleurodont tooth per jaw quadrant (blue shades). Hatchling skulls of two representative species are shown (with indicated positions in the plot). The black arrow indicates the relationship between increasing number of pleurodont teeth and decreased acrodont tooth width. (**H**) Scatter plot depicting individual tooth width in the Iguania fossil record, including Acrodonta and non-Acrodonta species showing different number of pleurodont teeth per jaw quadrant (red shades). Note again the relationship between increasing number of pleurodont teeth and decreased acrodont tooth width. Scale bars, 100 μm (B), 50 μm (G), and 1 cm (F).

To confirm these preliminary proliferation data, we next used two complementary approaches based on different M phase proliferation markers to quantify and obtain a more comprehensive understanding of dental proliferation pattern. First, we validated increased proliferation in mutant tissues using 3D reconstruction models of entire tooth germs stained by IHC with phosphorylated histone H3 (phospho-H3) (Fig. 3, F and G, and fig. S2E). Second, and because of the difficulty of obtaining tooth germ replicates at exact DL/initiation stage in vivo, we used an alternative ex vivo setting to allow quantification of cell proliferation through 5-bromo-2′-deoxyuridine (BrdU) incorporation in the same sample under different conditions (Fig. 4A). We further took advantage of the pharmacological inhibitor BAY 11-7082, which blocks EDA-mediated IκBα phosphorylation and downstream activation of NF-κB signaling in ectodermal tissues (*53*), to test and confirm the proliferative effect of EDA inhibition on intact wild-type dentition. Quantification of BrdU incorporation in anterior dentary tooth germs cultured for 1 week with BAY 11-7082 or vehicle control reveals an average of 12-fold rise in cell proliferation in the presence of the inhibitor (Fig. 4, B to D), an effect directly reflected by a significant increase in the tooth area of cultures (Fig. 4E). Even more notable, upon treatment with a high concentration of BAY 11-7082, some samples show massive proliferation resulting in completely defective tooth shape and abnormal cellular structure, also indicating a dose-dependent effect of NF-κB/EDA inhibition on cell proliferation and normal odontogenesis (fig. S2F). Changes in EDA dosage have also been suggested to play a role in the evolution of distinct organs, such as defensive armor plates of stickleback fish, suggesting that such a mechanism could be highly conserved in affecting the phenotypes of ectodermal organs (*3*). Together, both in vivo and ex vivo data show the role of EDA signaling in regulating the proliferation pattern of all different tooth types either directly or indirectly, thus corroborating both the size phenotype and transcriptome profile of scaleless *P. vitticeps* dentition. Changes in cell cycle gene expression and proliferation pattern are further reflected by phenotypic variation in tooth identity, as particularly well illustrated by the apparent transformation at both morphological and molecular levels of *EDA*-deficient anteriormost dentary teeth, highlighting the importance of proliferation index in determining pleurodont versus acrodont dentition.

### Predictive models of dental formula

Given the dose-dependent impact of EDA on tooth size and dental formula in *P. vitticeps* and the known variation in dentition patterns across Acrodonta, we wished to test whether species with distinct dental formulae could exhibit differences in EDA signaling level. For this purpose, we investigated *EDA* expression pattern in the homodont veiled chameleon (*Chamaeleo calyptratus*), which totally lacks anterior pleurodont teeth. A reduced gene expression was observed in the embryonic DL and dental papilla of chameleon tissues, when compared with the heterodont *P. vitticeps*, suggesting a putative evolutionary role for EDA signaling level in the regulation of dental formula (Fig. 4F). This hypothesis is further supported by the phenotype of our scaleless *P. vitticeps* model that replicates some of the dental diversity seen both in chameleons and agamid lizards, including lack of pleurodont teeth and wider acrodont teeth. Specifically, these relationships suggest that a general increase in tooth size, possibly through alteration of EDA signaling level, could lead to reduced formation of anterior pleurodont teeth.

To validate this prediction, we next assessed whether tooth size could be used as a general factor to predict variations in pleurodont tooth number within Acrodonta. We initially explored several acrodont lizards showing dental heterogeneity in acrodont/pleurodont pattern by focusing on the hatchling stage to eliminate postnatal changes in adult dentition that could emerge from tooth replacement, tooth addition, and/or tooth wear in some of the studied species. Of note, investigation of a few surviving adult scaleless *P. vitticeps* showed the possible emergence of some pleurodont teeth as the jaw grew in size postnatally, further indicating that the dental pattern is largely affected but not exclusively determined by embryonic processes. Our sampling at the hatchling stage was limited to the few available oviparous species with established breeding conditions and/or collection records (table S3). We detected a significantly higher tooth width in homodont species lacking caniniform pleurodont teeth, including chameleons but also *Uromastyx*, when compared with heterodont species showing a mix of acrodont and pleurodont dentition (Fig. 4G). Notably, analysis of individual species with either one or more than one pleurodont tooth per quadrant at hatchling stage indicates an inverse correlation between tooth width and pleurodont tooth number, regardless of family relationships (Fig. 4G), thus supporting the potential of tooth measurement in predicting a particular dental formula condition.

Dental implantation is widely considered an important character for identifying and interpreting extant species and fossil material, especially in vertebrate taxa that exhibit a variety of dental implantations and/or scarce fossil records. In lepidosaurs, the underestimated complexity of dental evolution is particularly evident from some extinct acrodontans, priscagamids, and rhynchocephalians with intermediate, not fully acrodont dentition recently identified in new geographical areas (*10*, *14*, *15*, *22*, *54*). Furthermore, similar transitional acrodont implantation condition has also been observed in other reptilian lineages such as Captorhinidae, one of the earliest and most basal reptile families (*16*), further highlighting the key importance of underexplored dental features such as tooth formula and implantation in illuminating evolution of vertebrate dentitions. Because of the simplicity and potential multilineage application of our prediction model, we extended our measurement analysis to a panel of extinct iguanian lizards showing a large variety of well-preserved dentary dentitions (table S4), with the goal of testing whether the identified developmental basis associated with tooth identity could be extrapolated to fossil taxa in one of the most diverse groups of lizards (*55*). Similar to living taxa, fossil dentitions unambiguously show a clear inverse correlation between tooth width and pleurodont tooth number (Fig. 4H), thus confirming the value of our model for more informed taxonomic, phylogenetic, developmental, and ecological interpretations of early dentitions at least within reptile lineages.

## DISCUSSION

Our characterization of the effect of *EDA* deficiency in squamate dentitions uncovers new critical functions of this pathway in determining underexplored vertebrate dental characters such as tooth identity and formula. To our knowledge, the scaleless lizard model presented here is the first known example of gene mutation resulting in a marked positional change of tooth identity in vertebrates. These findings raise intriguing questions on the fundamental mechanisms of heterondonty and jaw patterning in vertebrates, as tooth identity in mouse has been proposed to arise from a complex “homeobox code model” based on gene expression pattern (*56*). Our mutant lizard data suggest that the determination of pleurodont-acrodont heterodont dentitions can be achieved by a more flexible mechanism, where a simple modulation of EDA signaling has major consequences on tooth formula, thus providing new perspectives on vertebrate tooth formula patterning. Although further investigation, including in jaw primordium, should provide key information on the existence or not of dental prepattern and tooth positional information in Lepidosauria, the dental pattern could be further regulated by a simple reaction-diffusion model, where the size and growth of developing tooth germs affect the initiation of adjacent teeth. In this scenario, because of the delayed formation of pleurodont versus acrodont dentition [this study and (*36*)], the development of larger acrodont tooth germs as observed in mutant *P. vitticeps* could inhibit the initiation of later-developing pleurodont teeth and take over the pleurodont position, thus resulting in a functional pleurodont-acrodont transformation anteriorly. The observation of tooth development arrest at initiation stage in some extreme cases of mutant pleurodont positions further supports the potential existence of inhibitory mechanisms.

Regardless of mechanism, the identified effect of EDA signaling on lizard dental pattern is compatible with the importance of morphoregulation and fine-tuning of conserved pathways in tooth evolution, as previously shown for BMP signaling (*48*, *57*). Such implication of morphoregulation could have been expected from the variability of dental patterns observed in nonmammalian lineages, as best exemplified by the mixed or even intermediate dentition types present in Lepidosauria. Furthermore, our complementary analyses on multiple species show how such a simple change in the activity of one signaling pathway could be used to predict the mechanisms associated with particular dental implantation conditions at large scale. Together, our work demonstrates that the origin and diversification of dental implantation modes, long a focus of multiple fields including paleontology, can now be approached through a combination of evo-devo approaches, including both the fossil record as well as morphological, molecular, and experimental data on extant animals, thus providing the most complete possible picture of evolutionary patterns and transitions in vertebrate dentitions.

## MATERIALS AND METHODS

### Sample collection

All embryonic and postnatal stages of bearded dragon (*P. vitticeps*) and veiled chameleon (*C. calyptratus*) lizards were obtained from reptile colonies at the University of Helsinki and/or from private breeders. For embryonic stages, fertilized eggs were incubated on a moistened vermiculite substrate at 29.5°C (*P. vitticeps*) or 26.5°C (*C. calyptratus*), and embryos were removed at regular days postoviposition (dpo) to obtain stages of interest. Embryos were staged on the basis of their external morphology according to complete developmental tables available for these species (*27*, *28*). The majority of homozygous (*Sca*/*Sca*) and heterozygous (*Sca/+*) mutant lizards were obtained through heterozygous breedings, and additional breedings of heterozygous males with wild-type females were performed to bypass the reduced fertility of adult mutants and increase the number of *Sca/+* embryos in some experiments. All genotyping was performed using polymerase chain reaction with extracted DNA samples from tail tissues and the following primers: forward primer (fp), CTGAATGACTGGTCTCGCATCA; reverse primer (rp) wild type, CCAGCCTAATCTGGACATGACT; and rp *Sca*, ATCCACACAGTTTGGTTCACCT (*26*). Data on additional iguanian lizard species, including hatchling or adult and fossil specimens, were sampled from our previous work (*58*), specialized retailers, the published literature, as well as from publicly available Digital Morphology (DigiMorph; www.digimorph.org) and MorphoSource (www.MorphoSource.org) databases (tables S1, S3, and S4). All reptile captive breedings and experiments were done following international standards and were approved by the Laboratory Animal Centre of the University of Helsinki and/or the National Animal Experiment Board in Finland (license numbers ESLH-2007-07445/ym-23, ESAVI/7484/04.10.07/2016, and ESAVI/13139/04.10.05/2017).

### CT scanning and 3D volume rendering

CT scans of *P. vitticeps* and *C. calyptratus* specimens were performed at the University of Helsinki using Scyscan 1272 instrument (Bruker, Belgium). Samples were initially fixed overnight or longer, depending on the sample size, in 4% paraformaldehyde (PFA) at 4°C and dehydrated in an increasing alcohol series. Scanning parameters were as described before (*27*, *36*) with some adjustment depending on specimen age and scan purpose. All CT scans were reconstructed using NRecon 1.6.10.4, 1.7.0.4, or 1.7.4.2 software (Bruker), and 3D volume rendering as well as segmentation were done manually using the software Amira 5.5.0 (Thermo Fisher Scientific, USA). Some CT scans from adult Acrodonta specimens were also obtained from DigiMorph and MorphoSource databases (table S1).

### Analysis of dental parameters

Phenotypic analysis of dental features in *P. vitticeps* at hatchling and early postnatal stages was performed using CT scan skull data corresponding to 8 wild-type, 12 *Sca*/+, and 6 *Sca*/*Sca P. vitticeps* samples. For assessment of acrodont tooth size, we measured the tooth base width (tooth-bone attachment) of each individual tooth in labial view for the six posteriormost acrodont teeth of all jaw quadrants from each sample. This method was chosen on the basis of the minimum number of apparent acrodont teeth observed among all samples, but also to avoid any potential bias that could be linked to the typical reduced size of the anteriormost teeth in some samples (including wild type) showing more than six teeth. The mean width of all teeth on dentary or maxillary bones was then calculated separately and used for statistical analysis. For assessment of tooth number, we calculated the total number of teeth on either the maxillary or dentary bones, and potential variation in animal head size was corrected by normalization with use of the anterior-posterior skull length, defined as the distance between the anteriormost tip of the premaxilla and the central point superior to the foramen magnum. No major differences in skull shape or size were detected between wild-type and mutant *P. vitticeps* samples (fig. S1B). All measurements were made using the 3D measuring tool on Amira 5.5.0. Comparative assessment of tooth size in hatchling and fossil iguanian lizards showing acrodont or a mix of acrodont/pleurodont teeth was performed using CT scan data or published 2D high-quality photographs of well-preserved dentary bones and dentitions (tables S3 and S4). Tooth width in labial view was initially measured as above for individual posterior acrodont teeth available in each specimen and then normalized to the total length of the respective tooth-bearing maxillary (for hatchlings) or dentary (for fossils) parts, depending on the availability and/or integrity of the samples. The mean of all teeth was then calculated as above. Statistical significance for all measurements was calculated using Student’s *t* test in Microsoft Excel, and significance was determined at *P* value <0.05.

### Histology and IHC on paraffin sections

Following dissection, tissues were fixed overnight in 4% PFA at 4°C, and late embryonic stages showing mineralization were decalcified for 1 to 12 weeks, depending on sample size, in 20% EDTA. Tissue processing, paraffin sectioning (at 5 μm), and hematoxylin and eosin staining of dental tissues were performed as described before (*36*). IHC fluorescence staining was performed as described (*26*), using overnight incubation at 4°C with primary antibodies known to recognize reptile and/or chicken epitopes: PCNA (1:300, mouse monoclonal, BioLegend, catalog no. 307901, Research Resource Identifier (RRID): AB_314691), BrdU (1:200, rat monoclonal, Abcam, catalog no. ab6326, RRID: AB_305426), phospho-H3 (1:200, rabbit polyclonal, Abcam, catalog no. ab5176, RRID: AB_304763), or SRY-box 2 (SOX2; 1:200, rabbit polyclonal, catalog no. ab97959, RRID: AB_2341193). Last, incubation with Alexa Fluor–conjugated secondary antibodies (Alexa Fluor-488 or Alexa Fluor-568, Thermo Fisher Scientific) was performed for 1 hour at room temperature (RT), and slides were mounted with Fluoroshield mounting medium (Sigma-Aldrich) containing 4′,6′-diamidino-2-phenylindole (DAPI). Images were acquired either with a Nikon Eclipse 90i widefield microscope and Nikon NIS-Elements Advanced Research 4.30.01 software or with a Leica DM5000B widefield microscope and Leica Application Suite X (LAS X) 3.4.2 12.4.18 software. Images were processed with Fiji package (*59*) and/or Adobe Photoshop CC using levels adjustment.

### 3D volume rendering and cell quantification

3D models of entire tooth germs were obtained from serial frontal or sagittal sections stained with Phospho-H3 IHC. Raw images were acquired with a Leica DM5000B widefield microscope and Leica Application Suite X (LAS X) 3.4.2 12.4.18 software before conversion into TIFF format using Fiji package (*59*). TIFF files were then cropped and opened on Amira 5.5.0 to generate image stacks separated into blue (DAPI) and red (phospho-H3) channels. Phospho-H3–positive cells were segmented from the red channel stacks using signal intensity selection, and dental epithelium was manually segmented from the blue channel stacks. The two selections were then overlaid to generate 3D tooth germs. For quantification of phospho-H3+ cells, the total number of positive cells from an equal number of serial sections from 32 dpo WT (*n* = 3) and *Sca*/+ (*n* = 4) anterior dentary tooth germs was counted and normalized to the tooth volume (in cubic millimeters) as determined by measuring tooth areas on Fiji. Statistical significance was calculated using Student’s *t* test in Microsoft Excel, and significance was determined at *P* value <0.05.

### In situ hybridization

Digoxigenin (DIG)–labeled antisense riboprobes corresponding to *P. vitticeps ALX1* (fp, CTGTCTCCCGTGAAAGGCAT; rp, TAACAGAAGTGGGTGACTGCC), *CCNB1* (fp, TGACCACTCGGAAAGCCTCA; rp, GCGTCCACTCTCCCTCATCA), *EDA* (fp, TCTTCAAGGCCAAGGATCAGC; rp, CTGAAGTCGGAAACCACCCC), *EDAR* (fp, GCCTGGGAGAATGCAAATGG; rp, CACAGCTCTGGAGTCCCTTG), *NF*Κ*BIA* (fp, AAGGACGAGGAGTACGAGAAC; rp, GCCTCAGCTGTTCCTGTATGA), and *SIX3* (fp, TGCCCACGCTCAACTTTTC; rp, CCGCCGAA CTGTGAGTAGGA) transcripts were designed on the basis of publicly available *P. vitticeps* genome sequence (*60*). ISH was performed on paraffin sections as described (*61*) using a hybridization temperature of 63°C. Following hybridization, sections were washed and blocked from nonspecific antibody binding with blocking solution (2% Roche blocking reagent and 5% sheep serum) before incubating overnight at 4°C (or 1 hour at RT) with anti-DIG antibodies conjugated to alkaline phosphatase (1:2000, sheep polyclonal, Sigma-Aldrich, catalog no. 11093274910, RRID: AB_2734716). A staining solution containing 5-bromo-4-chloro-3-indolyl phosphate and nitro blue tetrazolium was applied for 1 to 3 days at RT to visualize hybridization. Last, slides were mounted using Dako Faramount aqueous medium (Agilent) and imaged with an Olympus AX70 microscope. Images were processed with Adobe Photoshop CC, and ISH staining was enhanced for visualization by fake coloring the selected blue pixels with the “Select colour range” and “Brush” tools.

### RNAscope ISH assay

RNAscope probes for *P. vitticeps EDA-A1* and *EDAR* were designed by ACDbio (sequence available from the company upon request), and all reagents were directly purchased from ACDbio. All tissue processing steps were performed according to the manufacturer’s instructions and as described for murine dentition (*62*). For pretreatments, paraffin sections were preheated at 60°C, deparaffinized and rehydrated, and dried again at 60°C. Sections were then treated with hydrogen peroxide for 10 min and incubated in target retrieval solution at 85°C for 30 min. Last, the sections were dehydrated in absolute ethanol and dried for 30 min at 60°C. For counterstaining, slides were incubated in 50% hematoxylin solution for 30 s. Stained slides were imaged and processed as described above for ISH.

### Laser microdissection, RNA sequencing, and transcriptome analysis

We analyzed the transcriptome of laser-microdissected dental tissues corresponding to posterior (acrodont position in wild type) and anteriormost (pleurodont position) dentition at hatchling SDL stage (corresponding to embryonic DL/initiation tooth stage) in three wild-type and three *Sca*/+ *P. vitticeps*. Tissue processing and sectioning, laser capture microdissection of epithelial and mesenchymal tissues using a Zeiss PALM Microbeam device equipped with a Zeiss AxioCam IC camera and PALM Robo software, and RNA extraction were performed as described before (*36*). Because of the nature of the experiment, the exact dental phenotype of *Sca*/+ samples could not be determined before tissue processing, but rough visual assessment before sectioning suggested that all three mutant samples had a representative abnormal dentary phenotype anteriorly. RNA integrity and concentration were assessed using Bioanalyzer RNA 6000 pico assays (Agilent). Ribosomal RNA (rRNA) depleted libraries were produced using the Nugen Ovation SoLo kit, and paired-end sequencing was done on a NextSeq 500 platform (Illumina, USA). Initial quality trimming, length filtering, and adapter sequence removal of reads were done as before (*36*). The STAR software (*63*) was used for sequence alignment to the *P. vitticeps* genome (*60*) and for generating count data. Count data were subsequently used for normalization, and differential gene expression analysis was conducted with edgeR (*64*) using three biological replicates. Genes with false discovery rate–corrected *P* values <0.05 were considered differentially expressed (table S2). MDS and PC analyses were carried out on normalized count data using the ClustVis v1 and iDEP v0.91 web applications for differential expression and pathway analysis (*65*, *66*). GO analyses, including GO term enrichment analysis of PC rotation, were performed using biological process GO terms in iDEP v0.91.

### Tooth explant cultures

The mandibles from six wild-type embryos corresponding to tooth DL/initiation stage (32 dpo) were dissected and divided vertically into left and right dentary components with a scalpel to obtain both control and test tissues from the same individuals (Fig. 4A). Each dentary was placed onto Millicell 0.4-μm cell culture inserts (Millipore) in six-well plates filled with DMEM/F12 medium (Gibco) containing 10% fetal calf serum, penicillin-streptomycin (100 U/ml; Gibco), 1:100 nystatin (Gibco), 1:100 amphotericin B (Gibco), and ascorbic acid (0.1 mg/ml). Dentaries were cultured at the air-liquid interface for 7 days at 35°C with 5% CO_2_ in the presence of 20 or 40 μM BAY 11-7082 (Abcam, catalog no. ab141228) dissolved in 100% ethanol or the corresponding volume of vehicle (100% ethanol). To assess proliferation, BrdU (Sigma-Aldrich) was added to the culture medium at 10 μM for 24 hours before the end of the cultures. Culture samples were then fixed in 4% PFA overnight, processed into paraffin blocks, and sectioned at 5 μm. BrdU IHC was performed on sections as described above. Cell counts of both proliferative BrdU-positive cells and total number of cells (based on DAPI staining) were done on three to five sections corresponding to each individual tooth using the “Count” tool on Adobe Photoshop CC, and BrdU/DAPI ratio was used to determine the mitotic index. Tooth area was calculated through manual selection of the tooth germ (including the enamel organ and DL) and analysis of the size of the selection using the “Analyze particles” tool of ImageJ program. Statistical significance was calculated using Student’s *t* test in Microsoft Excel, and significance was determined at *P* value <0.05.

## Supplementary Materials

### This PDF file includes:

Figs. S1 and S2 Tables S1, S3, and S4 References

## Notes

View/request a protocol for this paper from *Bio-protocol*.
