# Supplementary material for: The developmental origins of heterodonty and acrodonty as revealed by reptile dentitions
Source: Sci Adv. 2021 Dec 17;7(51):eabj7912. doi: 10.1126/sciadv.abj7912 (PMC8682985; doi:10.1126/sciadv.abj7912)
Supplement: Supplementary file 1 — Figs. S1 and S2 Tables S1, S3, and S4 References [file sciadv.abj7912_sm.pdf]

Science Advances
AAAS

Supplementary Materials for

The developmental origins of heterodonty and acrodonty as revealed by reptile dentitions

Lotta Salomies, Julia Eymann, Joni Ollonen, Imran Khan, Nicolas Di-Poï*

*Corresponding author. Email: nicolas.di-poi@helsinki.fi

Published 17 December 2021, *Sci. Adv.* **7**, eabj7912 (2021)
DOI: 10.1126/sciadv.abj7912

The PDF file includes:

Figs. S1 and S2
Tables S1, S3, and S4
References

Other Supplementary Material for this manuscript includes the following:

Table S2

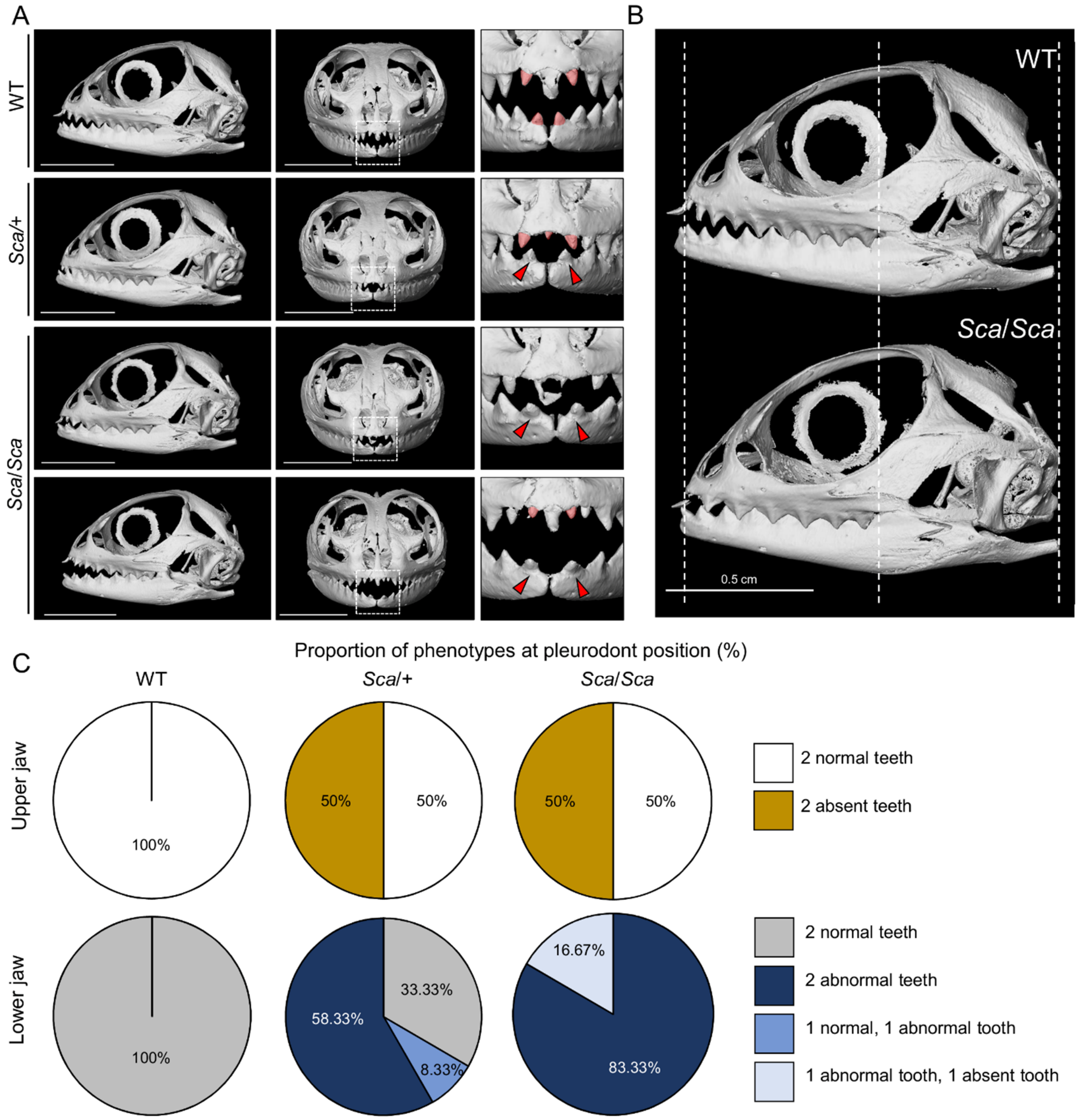

Fig. S1. Phenotypic characterization of mutant scaleless *P. vitticeps* dentition. (A) 3D-rendered skulls of littermate wild-type (WT), heterozygous scaleless (*Sca*/+), and homozygous scaleless (*Sca*/*Sca*) *P. vitticeps* at hatchling stage in lateral (left panels) and frontal (middle and right panels) views. Right panels show closeups of frontal views. Morphologically normal pleurodont teeth are highlighted in red, whereas large, acrodont-like teeth at anteriormost dentary position identified in mutants are indicated by red arrowheads. **(B)** Alignment of hatchling WT and *Sca*/*Sca* 3D-rendered skulls in scale, indicating no major variations in skull size and length. **(C)** Quantification of the various dental phenotypes seen in WT (n = 8), *Sca*/+ (n = 12), and *Sca*/*Sca* (n = 6) *P. vitticeps* at hatchling and early postnatal stage based on visual assessment from 3D-rendered skulls. Abnormal teeth only refer to the presence of large, acrodont-like tooth phenotype. Scale bars: 5 mm (A, B).

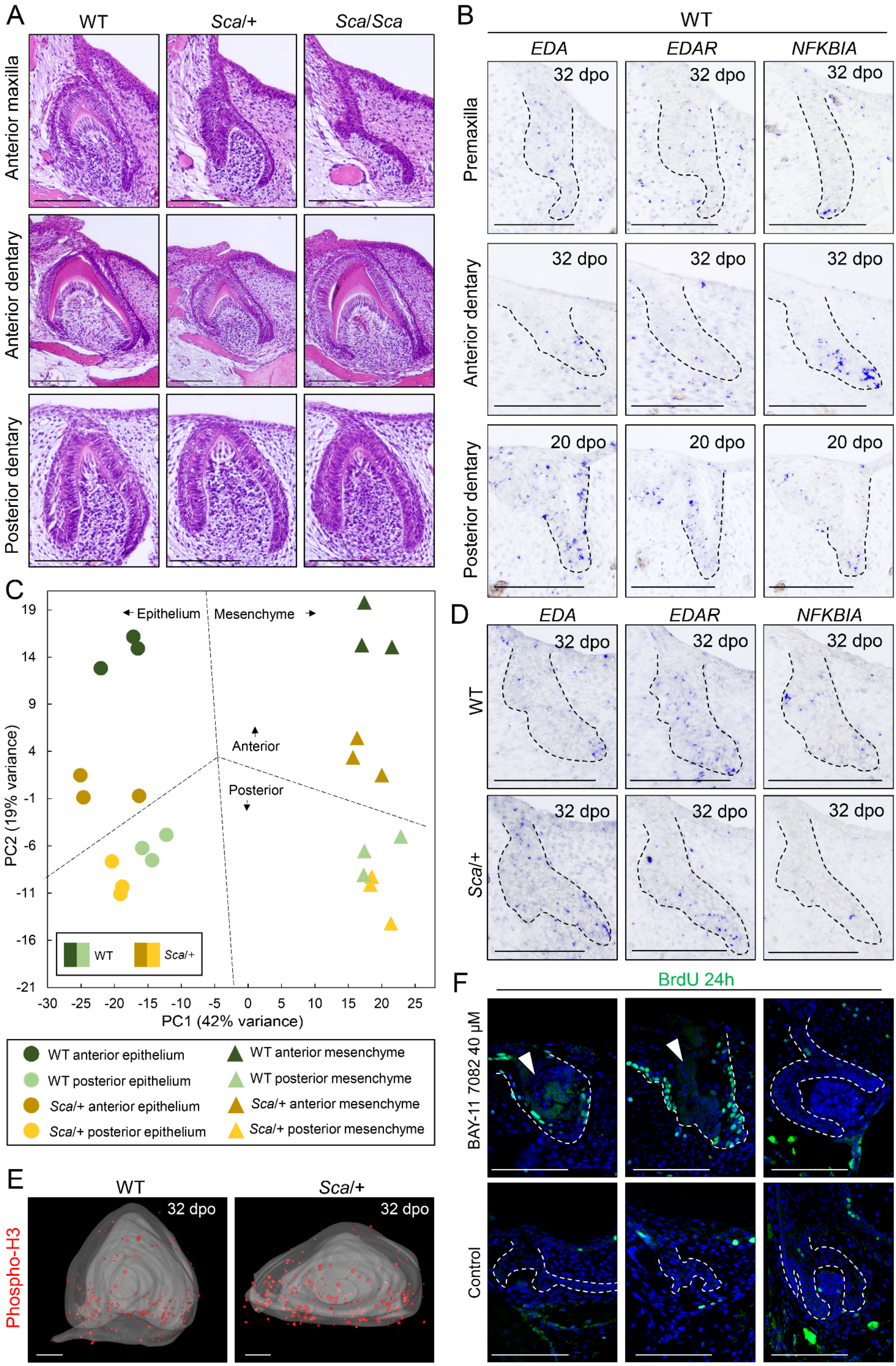

Fig. S2. Molecular characterization of mutant scaleless *P. vitticeps* dentition. (A) Hematoxylin and eosin (H&E)-stained sections of hatchling WT, *Sca*/+, and *Sca*/*Sca* *P. vitticeps* teeth from

premaxillary, anterior dentary, and posterior dentary positions. The *Sca/Sca* sample corresponds to extreme phenotype with total absence of visible teeth on the anterior maxilla (see Fig. S1C). **(B)** ISH of EDA, EDAR, and NFKBIA at DL/initiation stage in WT teeth from premaxillary, anterior dentary, and posterior dentary positions. Developmental stages are shown in dpo. The epithelium-mesenchyme junction is indicated by black dashed lines. **(C)** Principal component (PC) analysis of the same tooth transcriptome datasets at DL/initiation shown in Fig. 3B. Numbers in brackets indicate the percentage of variance explained by each of the main PC axes. **(D)** ISH of *EDA*, *EDAR*, and *NFKBIA* at DL/initiation stage in WT and Sca/+ teeth from anterior dentary position. Developmental stages are shown in dpo. The epithelium-mesenchyme junction is indicated by black dashed lines. **(E)** 3D reconstructed models of Phospho-H3 staining in posterior dentary tooth germs at mid-bell stage (32 dpo) from WT and *Sca*/+ embryos. **(F)** BrdU IHC on anterior dentary tooth germs cultured with either 40 mM BAY11-7082 or ethanol (control). Some samples show a distorted phenotype, high cell proliferation, and abnormal cell phenotype (white arrowheads).

Table S1. List of adult Acrodonta used in CT-scan analysis

Species	Family	Source (reference/database)
Agama hispida	Agaminae	*(58)*
Bronchocela jubata	Draconinae	*(58)*
Brookesia brygooi	Brookesiinae	DigiMorph
Chameleo calyptratus	Chamaeleonidae	This study
Hydrosaurus amboinensis	Hydrosaurinae	MorphoSource
Leiolepis belliana	Leiolepidinae	DigiMorph
Physignathus cocincinus	Amphibolurinae	This study
Uromastyx hardwicki	Uromastycinae	Digimorph

Table S2 (separate file)

Table S3. List of hatchling Acrodonta used in tooth size analysis

**Species**	**Family**	**Nber of pleurodont teeth per jaw quadrant**	**Normalized acrodont tooth width: mean ± SD (in mm)**	**Source (reference)**
Agama agama	Agamidae	1	0.110 ± 0.025	*(11)*
Amphibolurus muricatus	Agamidae	>1	0.081 ± 0.012	*(67)*
Bradypodion pumilum	Chamaeleonidae	0	0.120 ± 0.023	*(68)*
Chameleo calyptratus	Chamaeleonidae	0	0.112 ± 0.026	This study
Chamaeleo hoehnelli	Chamaeleonidae	0	0.127 ± 0.024	*(68)*
Ctenophorus nuchalis	Agamidae	1	0.105 ± 0.022	*(67)*
Furcifer pardalis	Chamaeleonidae	0	0.124 ± 0.037	This study
Gowidon longirostris	Agamidae	>1	0.092 ± 0.017	*(67)*
Pogona vitticeps	Agamidae	1	0.100 ± 0.028	This study
Uromastyx hardwicki	Agamidae	0	0.122 ± 0.038	*(26)*

Table S4. List of well-preserved fossil Iguania showing acrodont or a mix of acrodont/pleurodont dentition used in tooth size analysis

Species	Family (or clade)	Nber of pleurodont teeth per maxilla	Normalized acrodont tooth width: mean (SD) in mm	Source (reference)
Agama s.l.	Agamidae	2	0.099 ± 0.015	*(69)*
Chamaeleo caroliquarti	Chamaeleonidae	0	0.128 ± 0.044	*(70)*
Chamaeleo sp	Chamaeleonidae	0	0.125 ± 0.059	*(71)*
Gueragama sulamericana	Acrodonta	5	0.096 ± 0.007	*(14)*
Heredontagama borsukae	Priscagamidae	2-3	0.098 ± 0.036	*(72)*
Mimeosaurus crassus	Priscagamidae	0	0.122 ± 0.066	*(73)*
Priscagama gobiensis	Priscagamidae	6	0.089 ± 0.017	*(73)*
Tinosaurus doumuensis	Agamidae	4	0.094 ± 0.010	*(74)*
Tinosaurus europeocaenus	Agamidae	4	0.091 ± 0.033	*(75)*
Tinosaurus indicus	Agamidae	2-3	0.102 ± 0.042	*(72)*
Qianshanosaurus huangpuensis	Isodontosauridae	0	0.119 ± 0.017	*(74)*
Suratagama neeraae	Agamidae	6	0.082 ± 0.015	*(72)*
Vastanagama susanae	Agamidae	3	0.104 ± 0.042	*(72)*

REFERENCES AND NOTES

1. J. Jernvall, I. Thesleff, Tooth shape formation and tooth renewal: Evolving with the same signals. *Development* **139**, 3487–3497 (2012).

2. E. Harjunmaa, K. Seidel, T. Häkkinen, E. Renvoisé, I. J. Corfe, A. Kallonen, Z. Q. Zhang, A. R. Evans, M. L. Mikkola, I. Salazar-Ciudad, O. D. Klein, J. Jernvall, Replaying evolutionary transitions from the dental fossil record. *Nature* **512**, 44–48 (2014).

3. A. Sadier, L. Viriot, S. Pantalacci, V. Laudet, The ectodysplasin pathway: From diseases to adaptations. *Trends Genet.* **30**, 24–31 (2014).

4. H. Gomes Rodrigues, S. Renaud, C. Charles, Y. Le Poul, F. Solé, J. P. Aguilar, J. Michaux, P. Tafforeau, D. Headon, J. Jernvall, L. Viriot, Roles of dental development and adaptation in rodent evolution. *Nat. Commun.* **4**, 2504 (2013).

5. R. Peterková, M. Peterka, L. Viriot, H. Lesot, Development of the vestigial tooth primordia as part of mouse odontogenesis. *Connect. Tissue Res.* **43**, 120–128 (2002).

6. C. M. Brown, C. S. Vanburen, D. W. Larson, K. S. Brink, N. E. Campione, M. J. Vavrek, D. C. Evans, Tooth counts through growth in diapsid reptiles: Implications for interpreting individual and size-related variation in the fossil record. *J. Anat.* **226**, 322–333 (2015).

7. R. A. García, V. Zurriaguz, Histology of teeth and tooth attachment in titanosaurs (Dinosauria; Sauropoda). *Cretac. Res.* **57**, 248–256 (2016).

8. T. Davit-Béal, H. Chisaka, S. Delgado, J. Y. Sire, Amphibian teeth: Current knowledge, unanswered questions, and some directions for future research. *Biol. Rev. Camb. Philos. Soc.* **82**, 49–81 (2007).

9. M. M. Smith, G. J. Fraser, T. A. Mitsiadis, Dental lamina as source of odontogenic stem cells: Evolutionary origins and developmental control of tooth generation in gnathostomes. *J. Exp. Zool. Part B Mol. Dev. Evol.* **312**, 260–280 (2009).

10. T. J. C. Bertin, B. Thivichon-Prince, A. R. H. LeBlanc, M. W. Caldwell, L. Viriot, Current perspectives on tooth implantation, attachment, and replacement in amniota. *Front. Physiol.* **9**, 1630 (2018).

11. J. S. Cooper, D. F. G. Poole, R. Lawson, The dentition of agamid lizards with special reference to tooth replacement. *J. Zool.* **162**, 85–98 (1970).

12. S. E. Evans, G. V. R. Prasad, B. K. Manhas, Fossil lizards from the jurassic kota formation of India. *J. Vertebr. Paleontol.* **22**, 299–312 (2002).

13. S. A. Hocknull, Comparative maxillary and dentary morphology of the Australian dragons (Agamidae: Squamata): A framework for fossil identification. *Mem. Queensl. Museum* **48**, 125–145 (2002).
14. T. R. Simões, E. Wilner, M. W. Caldwell, L. C. Weinschütz, A. W. A. Kellner, A stem acrodontan lizard in the Cretaceous of Brazil revises early lizard evolution in Gondwana. *Nat. Commun.* **6**, 8149 (2015).
15. K. M. Jenkins, M. E. H. Jones, T. Zikmund, A. Boyde, J. D. Daza, A review of tooth implantation among rhynchocephalians (Lepidosauria). *J. Herpetol.* **51**, 300–306 (2017).
16. Y. Haridy, A. R. H. LeBlanc, R. R. Reisz, The Permian reptile *Opisthodontosaurus carrolli*: A model for acrodont tooth replacement and dental ontogeny. *J. Anat.* **232**, 371–382 (2018).
17. Y. Haridy, Histological analysis of post-eruption tooth wear adaptations, and ontogenetic changes in tooth implantation in the acrodontan squamate *Pogona vitticeps*. *PeerJ.* **6**, e5923 (2018).
18. B. Berkovitz, P. Shellis, *The Teeth of Non-Mammalian Vertebrates* (Elsevier, Amsterdam, 2017).
19. F. Lafuma, I. J. Corfe, J. Clavel, N. Di-Poï, Multiple evolutionary origins and losses of tooth complexity in squamates. *Nat. Commun.* **12**, 6001 (2021).
20. D. L. Mahler, M. Kearney, The palatal dentition in squamate reptiles: Morphology, development, attachment, and replacement. *Fieldiana Zool.* **108**, 1–61 (2006).
21. S. E. Evans, "The skull of Lepidosauria" in *Biology of the Reptilia* (Ithaca, NY, 2008), vol. 20, pp. 1–755.
22. M. Borsuk-Białynicka, The late cretaceous lizard *Pleurodontagama* and the origin of tooth permanency in Lepidosauria. *Acta Palaeontol. Pol.* **41**, 231–252 (1996).
23. S. E. Evans, M. Borsuk-Białynicka, A small lepidosauromorph reptile from the early Triassic of Poland. *Palaeontol. Pol.* **65**, 179–202 (2009).
24. H. Zaher, O. Rieppel, Tooth implantation and replacement in squamates, with special reference to mosasaur lizards and snakes. *Am. Museum Novit.* **3271**, 1–19 (1999).
25. J. S. Cooper, D. F. G. Poole, The dentition and dental tissues of the agamid lizard, *Uromastyx*. *J. Zool.* **169**, 85–100 (1973).
26. N. Di-Poï, M. C. Milinkovitch, The anatomical placode in reptile scale morphogenesis indicates shared ancestry among skin appendages in amniotes. *Sci. Adv.* **2**, e1600708 (2016).

27. J. Ollonen, F. O. Da Silva, K. Mahlow, N. Di-Poï, Skull development, ossification pattern, and adult shape in the emerging lizard model organism *Pogona vitticeps*: A comparative analysis with other squamates. *Front. Physiol.* **9**, 278 (2018).
28. R. E. Diaz Jr., N. A. Shylo, D. Roellig, M. Bronner, P. A. Trainor, Filling in the phylogenetic gaps: Induction, migration, and differentiation of neural crest cells in a squamate reptile, the veiled chameleon (*Chamaeleo calyptratus*). *Dev. Dyn.* **248**, 709–727 (2019).
29. C. Drögemüller, H. Kuiper, M. Peters, S. Guionaud, O. Distl, T. Leeb, Congenital hypotrichosis with anodontia in cattle: A genetic, clinical and histological analysis. *Vet. Dermatol.* **13**, 307–313 (2002).
30. P. F. Colosimo, K. E. Hosemann, S. Balabhadra, G. Villarreal, H. Dickson, J. Grimwood, J. Schmutz, R. M. Myers, D. Schluter, D. M. Kingsley, Widespread parallel evolution in sticklebacks by repeated fixation of ectodysplasin alleles. *Science* **307**, 1928–1933 (2005).
31. C. S. Barlund, E. G. Clark, T. Leeb, C. Drögemüller, C. W. Palmer, Congenital hypotrichosis and partial anodontia in a crossbred beef calf. *Can. Vet. J.* **48**, 612–614 (2007).
32. M. L. Casal, P. F. Jezyk, J. M. Greek, M. H. Goldschmidt, D. F. Patterson, X-linked ectodermal dysplasia in the dog. *J. Hered.* **88**, 513–517 (1997).
33. M. P. Harris, N. Rohner, H. Schwarz, S. Perathoner, P. Konstantinidis, C. Nüsslein-Volhard, Zebrafish *eda* and *edar* mutants reveal conserved and ancestral roles of ectodysplasin signaling in vertebrates. *PLOS Genet.* **4**, e1000206 (2008).
34. A. D. S. Atukorala, K. Inohaya, O. Baba, M. J. Tabata, R. A. R. K. Ratnayake, D. Abduweli, S. Kasugai, H. Mitani, Y. Takano, Scale and tooth phenotypes in medaka with a mutated ectodysplasin-a receptor: Implications for the evolutionary origin of oral and pharyngeal teeth. *Arch. Histol. Cytol.* **73**, 139–148 (2011).
35. N. M. O'Brown, B. R. Summers, F. C. Jones, S. D. Brady, D. M. Kingsley, A recurrent regulatory change underlying altered expression and Wnt response of the stickleback armor plates gene *EDA*. *eLife* **4**, e05290 (2015).
36. L. Salomies, J. Eymann, I. Khan, N. Di-Poï, The alternative regenerative strategy of bearded dragon unveils the key processes underlying vertebrate tooth renewal. *eLife* **8**, e47702 (2019).
37. J. Laurikkala, M. Mikkola, T. Mustonen, T. Åberg, P. Koppinen, J. Pispa, P. Nieminen, J. Galceran, R. Grosschedl, I. Thesleff, TNF signaling via the ligand-receptor pair ectodysplasin

and edar controls the function of epithelial signaling centers and is regulated by Wnt and activin during tooth organogenesis. *Dev. Biol.* **229**, 443–455 (2001).
38. K. M. Melstrom, The relationship between diet and tooth complexity in living dentigerous saurians. *J. Morphol.* **278**, 500–522 (2017).
39. S. Miard, R. Peterková, J. L. Vonesch, M. Peterka, J. V. Ruch, H. Lesot, Alterations in the incisor development in the Tabby mouse. *Int. J. Dev. Biol.* **43**, 517–529 (1999).
40. P. Kristenová-Cermáková, M. Peterka, S. Lisi, H. Lesot, R. Peterková, Postnatal lower jaw dentition in different phenotypes of tabby mice. *Connect. Tissue Res.* **43**, 283–288 (2002).
41. A. S. Tucker, D. J. Headon, J. M. Courtney, P. Overbeek, P. T. Sharpe, The activation level of the TNF family receptor, Edar, determines cusp number and tooth number during tooth development. *Dev. Biol.* **268**, 185–194 (2004).
42. J. Pispa, H. S. Jung, J. Jernvall, P. Kettunen, T. Mustonen, M. J. Tabata, J. Kere, I. Thesleff, Cusp patterning defect in Tabby mouse teeth and its partial rescue by FGF. *Dev. Biol.* **216**, 521–534 (1999).
43. A. S. Tucker, D. J. Headon, P. Schneider, B. M. Ferguson, P. Overbeek, J. Tschopp, P. T. Sharpe, Edar/Eda interactions regulate enamel knot formation in tooth morphogenesis. *Development* **127**, 4691–4700 (2000).
44. C. Charles, S. Pantalacci, P. Tafforeau, D. Headon, V. Laudet, L. Viriot, Distinct impacts of *Eda* and *Edar* loss of function on the mouse dentition. *PLOS ONE* **4**, e4985 (2009).
45. M. Landova Sulcova, O. Zahradnicek, J. Dumkova, H. Dosedelova, J. Krivanek, M. Hampl, M. Kavkova, T. Zikmund, M. Gregorovicova, D. Sedmera, J. Kaiser, A. S. Tucker, M. Buchtova, Developmental mechanisms driving complex tooth shape in reptiles. *Dev. Dyn.* **249**, 441–464 (2020).
46. P. N. Kantaputra, R. J. Gorlin, Double dens invaginatus of molarized maxillary central incisors, premolarization of maxillary lateral incisors, multituberculism of the mandibular incisors, canines and first premolar, and sensorineural hearing loss. *Clin. Dysmorphol.* **1**, 128–36 (1992).
47. A. S. Tucker, K. L. Matthews, P. T. Sharpe, Transformation of tooth type induced by inhibition of BMP signaling. *Science* **282**, 1136–1138 (1998).

48. P. M. Munne, S. Felszeghy, M. Jussila, M. Suomalainen, I. Thesleff, J. Jernvall, Splitting placodes: Effects of bone morphogenetic protein and Activin on the patterning and identity of mouse incisors. *Evol. Dev.* **12**, 383–392 (2010).
49. A. R. H. LeBlanc, I. Paparella, D. O. Lamoureux, M. R. Doschak, M. W. Caldwell, Tooth attachment and pleurodont implantation in lizards: Histology, development, and evolution. *J. Anat.* **238**, 1156–1178 (2021).
50. S. C. Sun, P. A. Ganchi, D. W. Ballard, W. C. Greene, NF-κB controls expression of inhibitor IκBα: Evidence for an inducible autoregulatory pathway. *Science* **259**, 1912–1915 (1993).
51. H. R. Dassule, P. Lewis, M. Bei, R. Maas, A. P. McMahon, Sonic hedgehog regulates growth and morphogenesis of the tooth. *Development* **127**, 4775–4785 (2000).
52. E. Juuri, M. Jussila, K. Seidel, S. Holmes, P. Wu, J. Richman, K. Heikinheimo, C.-M. Chuong, K. Arnold, K. Hochedlinger, O. Klein, F. Michon, I. Thesleff, Sox2 marks epithelial competence to generate teeth in mammals and reptiles. *Development* **140**, 1424–1432 (2013).
53. C. Mou, B. Jackson, P. Schneider, P. A. Overbeek, D. J. Headon. Generation of the primary hair follicle pattern. *Proc. Natl. Acad. Sci. U.S.A.* **103**, 9075–9080 (2006).
54. V. R. Alifanov, New acrodont lizards (Lacertilia) from the Middle Eocene of southern Mongolia. *Paleontol. J.* **43**, 675–685 (2009).
55. J. F. R. Tonini, K. H. Beard, R. B. Ferreira, W. Jetz, R. A. Pyron, Fully-sampled phylogenies of squamates reveal evolutionary patterns in threat status. *Biol. Conserv.* **204**, 23–31 (2016).
56. B. L. Thomas, P. T. Sharpe, Patterning of the murine dentition by homeobox genes. *Eur. J. Oral Sci.* **106**, 48–54 (1998).
57. M. V. Plikus, M. Zeichner-David, J.-A. Mayer, J. Reyna, P. Bringas, J. G. M. Thewissen, M. L. Snead, Y. Chai, C.-M. Chuong, Morphoregulation of teeth: Modulating the number, size, shape and differentiation by tuning Bmp activity. *Evol. Dev.* **7**, 440–457 (2005).
58. F. O. Da Silva, A. C. Fabre, Y. Savriama, J. Ollonen, K. Mahlow, A. Herrel, J. Müller, N. Di-Poï, The ecological origins of snakes as revealed by skull evolution. *Nat. Commun.* **9**, 376 (2018).
59. J. Schindelin, I. Arganda-Carreras, E. Frise, V. Kaynig, M. Longair, T. Pietzsch, S. Preibisch, C. Rueden, S. Saalfeld, B. Schmid, J.-Y. Tinevez, D. J. White, V. Hartenstein, K. Eliceiri, P.

Tomancak, A. Cardona, Fiji: An open-source platform for biological-image analysis. *Nat. Methods* **9**, 676–682 (2012).
60. A. Georges, Q. Li, J. Lian, D. O'Meally, J. Deakin, Z. Wang, P. Zhang, M. Fujita, H. R. Patel, C. E. Holleley, Y. Zhou, X. Zhang, K. Matsubara, P. Waters, J. A. M. Graves, S. D. Sarre, G. Zhang, High-coverage sequencing and annotated assembly of the genome of the Australian dragon lizard *Pogona vitticeps*. *Gigascience* **4**, 45 (2015).
61. J. Eymann, L. Salomies, S. Macrì, N. Di-Poï, Variations in the proliferative activity of the peripheral retina correlate with postnatal ocular growth in squamate reptiles. *J. Comp. Neurol.* **527**, 2356–2370 (2019).
62. M. Sanz-Navarro, K. Seidel, Z. Sun, L. Bertonnier-Brouty, B. A. Amendt, O. D. Klein, F. Michon, Plasticity within the niche ensures the maintenance of a *Sox2*$^+$ stem cell population in the mouse incisor. *Development* **145**, dev155929 (2018).
63. A. Dobin, C. A. Davis, F. Schlesinger, J. Drenkow, C. Zaleski, S. Jha, P. Batut, M. Chaisson, T. R. Gingeras, STAR: Ultrafast universal RNA-seq aligner. *Bioinformatics* **29**, 15–21 (2013).
64. M. D. Robinson, A. Oshlack, A scaling normalization method for differential expression analysis of RNA-seq data. *Genome Biol.* **11**, R25 (2010).
65. T. Metsalu, J. Vilo, ClustVis: A web tool for visualizing clustering of multivariate data using principal component analysis and heatmap. *Nucleic Acids Res.* **43**, W566–W570 (2015).
66. S. X. Ge, E. W. Son, R. Yao, iDEP: An integrated web application for differential expression and pathway analysis of RNA-Seq data. *BMC Bioinformatics* **19**, 534 (2018).
67. J. A. Gray, M. N. Hutchison, M. E. H. Jones, Exceptional disparity in Australian agamid lizards is a possible result of arrival into vacant niche. *Anat. Rec.* **302**, 1536–1543 (2019).
68. O. Rieppel, Studies on skeleton formation in reptiles. II. *Chamaeleo hoehnelii* (Squamata: Chamaeleoninae), with comments on the homology of carpal and tarsal bones. *Herpetologica* **49**, 66–78 (1993).
69. M. Delfino, T. Kotsakis, M. Arca, C. Tuveri, G. Pitruzzella, L. Rook, Agamid lizards from the Plio-Pleistocene of Sardinia (Italy) and an overview of the European fossil record of the family. *Geodiversitas* **30**, 641–656 (2008).
70. S. Moody, Z. Roček, *Chamaeleo caroliquarti* (Chamaeleonidae, Sauria): A new species from the Lower Miocene of Central Europe. *Věst. Ústř. Úst. geol.* **55**, 85–92 (1980).

71. O. Rieppel, A. Walker, I. Odhiambo, A preliminary report on a fossil chamaeleonine (Reptilia: Chamaeleoninae) skull from the Miocene of Kenya. *J. Herpetol.* **26**, 77–80 (1992).
72. R. S. Rana, M. Augé, A. Folie, K. D. Rose, K. Kumar, L. Singh, A. Sahni, T. Smith, High diversity of acrodontan lizards in the Early Eocene Vastan Lignite Mine of India. *Geol. Belg.* **16**, 290–301 (2013).
73. M. Borsuk-Białynicka, S. M. Moody, Priscagaminae, a new subfamily of the Agamidae (Sauria) from the Late Cretaceous of the Gobi Desert. *Acta Palaeontol. Pol.* **29**, 51–81 (1984).
74. D. Li-Ping, S. E. Evans, W. Yuan, Taxonomic revision of lizards from the Paleocene deposits of the Qianshan Basin, Anhui, China. *Vert. PalAs.* **54**, 243–268 (2016).
75. M. Augé, R. Smith, Les Agamidae (Reptilia, Squamata) du Paléogène d’Europe occidentale. *Belg. J. Zool.* **127**, 123–138 (1997).
